# Supplementary figures and images for: Antenatal corticosteroids for impending late preterm (34-36+6 weeks) deliveries—A systematic review and meta-analysis of RCTs
Source: PLoS One. 2021 Mar 22;16(3):e0248774. doi: 10.1371/journal.pone.0248774 (PMC7984612; doi:10.1371/journal.pone.0248774)

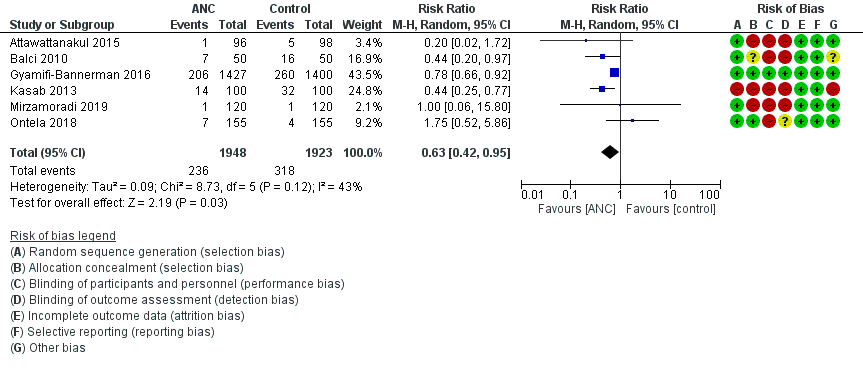

Supplement: S1 Fig — (TIF) [file pone.0248774.s001.tif]

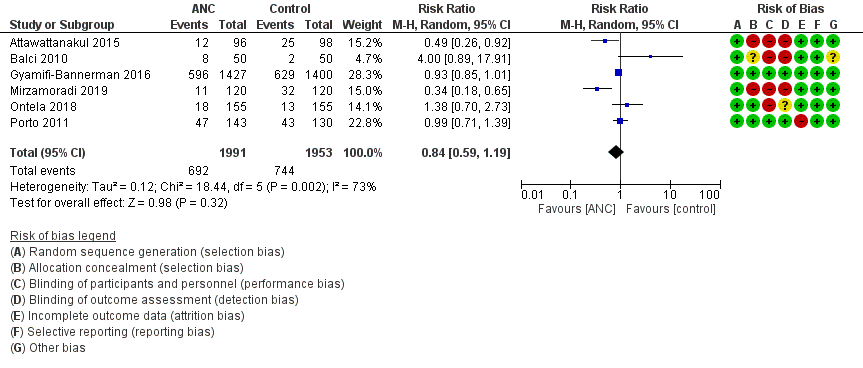

Supplement: S2 Fig — (TIF) [file pone.0248774.s002.tif]

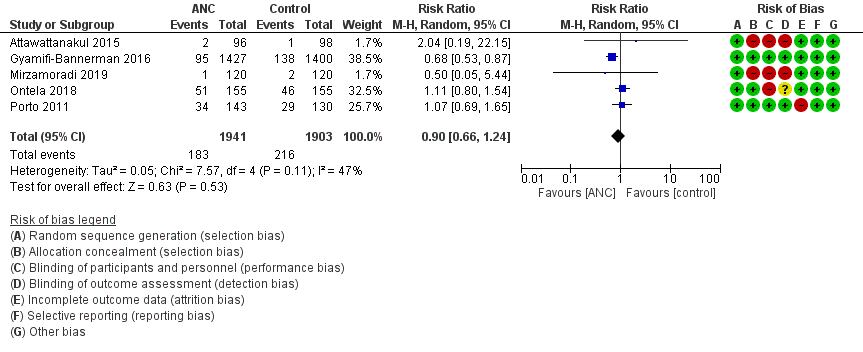

Supplement: S3 Fig — (TIF) [file pone.0248774.s003.tif]

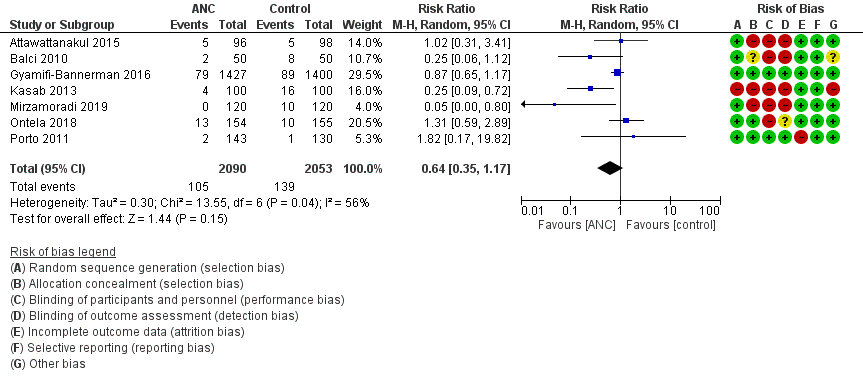

Supplement: S4 Fig — (TIF) [file pone.0248774.s004.tif]

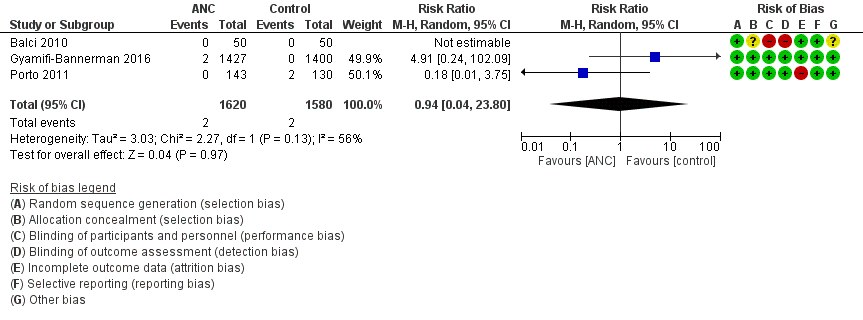

Supplement: S5 Fig — (TIF) [file pone.0248774.s005.tif]

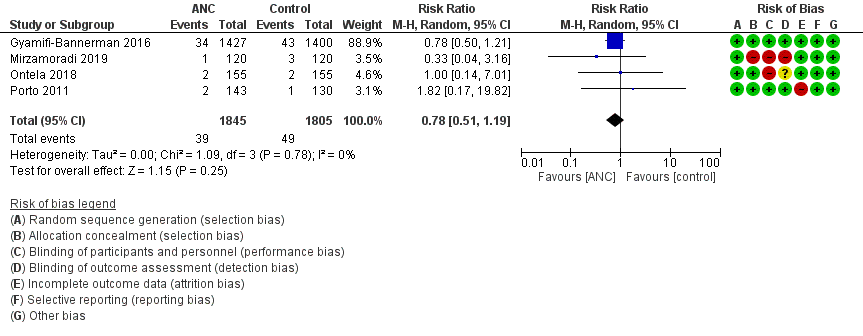

Supplement: S6 Fig — (TIF) [file pone.0248774.s006.tif]

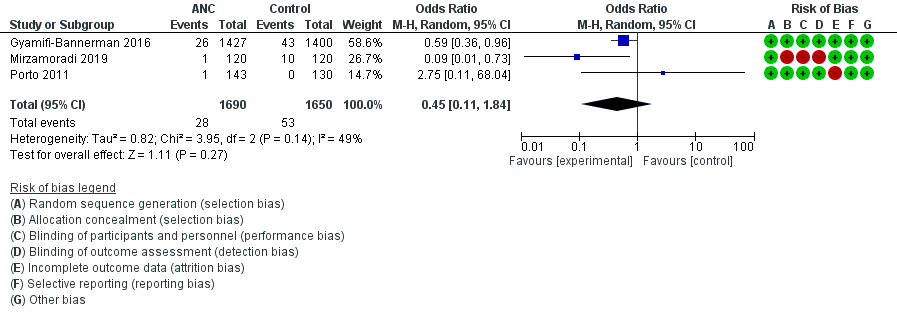

Supplement: S7 Fig — (TIF) [file pone.0248774.s007.tif]
