# Supplementary material for: Antenatal corticosteroids for impending late preterm (34-36+6 weeks) deliveries—A systematic review and meta-analysis of RCTs
Source: PLoS One. 2021 Mar 22;16(3):e0248774. doi: 10.1371/journal.pone.0248774 (PMC7984612; doi:10.1371/journal.pone.0248774)
Supplement: S1 Table — (DOCX) [file pone.0248774.s008.docx]

**S1 Table: Sensitivity analysis of the studies carrying low risk of bias**

| **Outcome**  **Effect of ANC on** | **RCTs with low ROB** | **Number of participants** | **Results** |
| --- | --- | --- | --- |
| **Any respiratory support** | (1) Gyamifi- Bannerman et al  (2) Porto et al | N: 3100  ANC:1570  C:1530 | RR 0.83  (0.64 to 1.08)  I^2^=28%  P=0.16 |
| **Respiratory distress syndrome** | (1) Gyamifi- Bannerman et al  (2) Porto et al | N: 3100  ANC:1570  C:1530 | RR 0.88  (0.66 to 1.18)  I^2^=0%  P=0.39 |
| **Transient tachypnoea of newborn** | (1) Gyamifi- Bannerman et al  (2) Porto et al | N: 3100  ANC:1570  C:1530 | RR 0.82  (0.53 to 1.27)  I2=69%  P=0.38 |
| **Hypoglycemia** | (1) Gyamifi- Bannerman et al  (2) Porto et al | N: 3100  ANC:1570  C:1530 | **RR 1.60**  **(1.37 to 1.86)**  **I^2^=0%**  **P<0.00001** |
| **Need for resus at birth** | (1) Gyamifi- Bannerman et al | N: 2827  ANC: 1427  C:1400 | **RR 0.78**  **(0.66 to 0.92)**  **P=0.003** |
| **Mortality** | (1) Gyamifi- Bannerman et al  (2) Porto et al | N: 3100  ANC: 1570  C:1530 | RR 0.94  (0.04 to 23.80)  I^2^=56%  P=0.97 |
| **Admission to NICU** | (1) Gyamifi- Bannerman et al  (2) Porto et al | N: 3100  ANC: 1570  C:1530 | RR 0.93  (0.86 to 1.01)  I^2^=0%  P=0.10 |
| **Need for mechanical ventilation** | (1) Gyamifi- Bannerman et al  (2) Porto et al | N: 3100  ANC: 1570  C:1530 | RR 0.80  (0.52 to 1.23)  I^2^=0%  P=0.31 |
| **Need for Surfactant** | (1) Gyamifi- Bannerman et al  (2) Porto et al | N: 3100  ANC: 1570  C:1530 | **RR 0.61**  **(0.37 to 0.99)**  **I^2^=0%**  **P=0.04** |

ANC: Antenatal corticosteroids, C: Control, CI: Confidence interval, RR: Relative risk
