# Supplementary material for: Antenatal corticosteroids for impending late preterm (34-36+6 weeks) deliveries—A systematic review and meta-analysis of RCTs
Source: PLoS One. 2021 Mar 22;16(3):e0248774. doi: 10.1371/journal.pone.0248774 (PMC7984612; doi:10.1371/journal.pone.0248774)
Supplement: S2 Table — (DOCX) [file pone.0248774.s009.docx]

**S2 Table: Comparison of the results based on method of analysis**

| **Outcome** | **Relative effect**  **RR (95% CI)** | |
| --- | --- | --- |
| **Effect of ANC on** | **Random Effects** | **Fixed effect** |
| **Any respiratory support** | RR 0.75  (0.58 to 0.97)  I^2^=22 | RR 0.76  (0.65 to 0.89)  I^2^=22 |
| **Hypoglycemia** | RR 1.61  (1.38 to 1.87)  I^2^=0 | RR 1.61  (1.39 to 1.87)  I^2^=0 |
| **Need for resus at birth** | RR 0.63  (0.42 to 0.95)  I^2^=43 | RR 0.73  (0.63 to 0.85)  I^2^=43 |
| **Mortality** | RR 0.94  (0.04 to 23.80)  I^2^=56% | RR 0.95  (0.20 to 4.58)  I^2^=56% |
| **Admission to NICU** | OR 0.84  (0.59 to 1.19)  I^2^=73% | OR 0.91  (0.84 to 0.99)  I^2^=73% |
| **Need for mechanical ventilation** | RR 0.78  (0.51 to 1.19)  I^2^=0 | RR 0.78  (0.51 to 1.18)  I^2^=0 |
| **Need for Surfactant** | RR 0.45  (0.11 to 1.84)  I^2^=49% | RR 0.51  (0.32 to 0.81)  I^2^=49% |
| **RDS** | RR 0.64  (0.35 to 1.17)  P=0.15  I^2^=56% | RR 0.75  (0.58 to 0.95)  P=0.02  I^2^=56% |
| **TTN** | RR 0.90  (0.66 to 1.24)  P=0.53  I^2^=47% | RR 0.83  (0.69 to 0.99)  P=0.04  I^2^=47% |

CI: Confidence interval, TTN: Transient tachypnoea of newborn, RDS: respiratory distress syndrome, RR: Relative risk
