# Supplementary material for: Antenatal corticosteroids for impending late preterm (34-36+6 weeks) deliveries—A systematic review and meta-analysis of RCTs
Source: PLoS One. 2021 Mar 22;16(3):e0248774. doi: 10.1371/journal.pone.0248774 (PMC7984612; doi:10.1371/journal.pone.0248774)
Supplement: S1 File — (DOCX) [file pone.0248774.s011.docx]

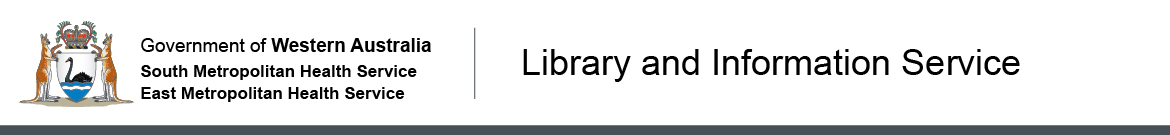


Documentation of literature searching provided in support the systematic review.

Title: **Effects of Antenatal corticosteroids in Late preterm neonates**

References from all sources searched were exported to an EndNote Library.

Search was done by Rhonda Mayberry with peer review from: Rogene McCoy & Marcia Powell

Search concept - PICO, SPIDER, ECLIPS(E) or other

P

I

C

Outcome

Inclusion criteria identified

All languages. All years

Databases Searched

**Database name: Medline**

Platform: Ovid

Database coverage (years): 1946 - 2019

Date of search 25 Nov 2019

Total number of references found 428 (409 if deduplicated in database)

Duplicates after deduplicating in Endnote 1

Actual number of refs 408

Search strategy included in appendix

======

**Database name: Embase**

Platform: Ovid

Database coverage (years): 1974 - 2019

Date of search 25 Nov 2019

Total number of references found 362 (351 if deduplicated in database)

Duplicates after deduplicating in Endnote 174

Actual number of refs 177

Search strategy included in appendix

======

**Database name: Web of Science Core Collection**

Platform: Clarivate Analytics

Database coverage (years): 1997 - 2019

Date of search 25 Nov 2019

Total number of references found 24

Duplicates after deduplicating in Endnote 12

Actual number of refs 12

Search strategy included in appendix

======

**Database name: Medline epub, Ahead of Print and In-Process & Other Non-indexed Citations**

Platform: Ovid

Database coverage (years): 1946 - 2019

Date of search 25 Nov 2019

Total number of references found 15

Duplicates after deduplicating in Endnote 6

Actual number of refs 9

Search strategy included in appendix

======

**Database name: PubMed** (for prepublication, in-process and non-Medline records)

Platform Internet

Date of search 25 Nov 2019

Total number of references found 17

Duplicates after deduplicating in Endnote 10

Actual number of refs 7

Search strategy included in appendix

======

**Web browser: Google Scholar**

Platform Internet

Date of search 25 / 26 Nov 2019

1940 results. Reviewed the first 300 records to find unique articles not identified in database searches.

Results : 14

Search strategy included in appendix

Detailed record of the search strategy that shows citation retrieval numbers at each step.

Database: Ovid MEDLINE(R) 1946 to Present with Daily Update (MESD)

Search Strategy:

--------------------------------------------------------------------------------

1 exp *Adrenal Cortex Hormones/tu (41379)

2 (corticosteroid* or betamethasone or celestone or dexamethasone).ti,ab. (138404)

3 (adrenal cortex adj hormone*).ti,ab. (632)

4 1 or 2 or 3 (165479)

5 Infant, Premature/ (52200)

6 Premature Birth/ (12845)

7 Pregnancy Trimester, Third/ (14344)

8 (prematur$ adj3 (birth$ or born or deliver$)).ti,ab. (13855)

9 5 or 6 or 7 or 8 (85264)

10 (preterm or preterms or pre term or pre terms).ti,ab. (62716)

11 (preemie* or premie or premies).ti,ab. (153)

12 (late preterm or near term or almost term or before term or early term).ti,ab. (7107)

13 10 or 11 or 12 (67788)

14 (newborn* or new born* or newly born* or baby or babies or infan*).ti,ab. (517681)

15 (neonat$ or neo nat$).ti,ab. (232289)

16 14 or 15 (656936)

17 *prenatal care/ (13873)

18 (antenatal or ante natal).ti,ab. (29661)

19 17 or 18 (39700)

20 16 or 19 (681508)

21 13 and 20 (47259)

22 9 or 21 (104876)

23 4 and 22 (2765)

24 limit 23 to randomized controlled trial (266)

25 limit 23 to clinical trial, phase iii (1)

26 "randomi?ed controlled trial".ti,ab,kw. (76752)

27 Randomized Controlled Trials as Topic/ (128629)

28 clinical trial, phase iii/ (15918)

29 (Phase 3 or phase3 or phase iii or P3 or Piii).ti,ab,kw. (55622)

30 26 or 27 or 28 or 29 (255374)

31 23 and 30 (241)

32 24 or 25 or 31 (446)

33 exp animals/ not humans.sh. (4645140)

34 32 not 33 (443)

35 34 not (letter or newspaper article or editorial).pt. (428)

36 remove duplicates from 35 (409)

********************

Database: Embase <1974 to 2019 Week 47>

Search Strategy:

--------------------------------------------------------------------------------

1 exp *corticosteroid/ (288605)

2 (corticosteroid* or betamethasone or celestone or dexamethasone).ti,ab. (218285)

3 (adrenal cortex adj hormone*).ti,ab. (124)

4 1 or 2 or 3 (421215)

5 dt.fs. or drug therapy/ (4292934)

6 4 and 5 (213551)

7 *Prematurity/ (45384)

8 *premature labor/ (15649)

9 third trimester pregnancy/ (27436)

10 exp *fetus maturity/ (1510)

11 (prematur$ adj3 (birth$ or born or deliver$)).ti,ab. (20826)

12 7 or 8 or 9 or 10 or 11 (102418)

13 (preterm or preterms or pre term or pre terms).ti,ab. (100254)

14 (preemie* or premie or premies).ti,ab. (249)

15 (late preterm or near term or almost term or before term or early term).ti,ab. (10395)

16 13 or 14 or 15 (107144)

17 (newborn* or new born* or newly born* or baby or babies or infan*).ti,ab. (660840)

18 (neonat$ or neo nat$).ti,ab. (330058)

19 17 or 18 (851143)

20 *prenatal care/ (11147)

21 (antenatal or ante natal).ti,ab. (47784)

22 20 or 21 (55050)

23 19 or 22 (882817)

24 16 and 23 (74418)

25 12 or 24 (143614)

26 6 and 25 (2448)

27 limit 26 to randomized controlled trial (263)

28 limit 26 to phase 3 clinical trial (3)

29 "randomi?ed controlled trial".ti,ab,kw. (127313)

30 Randomized Controlled Trials as Topic/ (105893)

31 phase 3 clinical trial/ (44150)

32 (Phase 3 or phase3 or phase iii or P3 or Piii).ti,ab,kw. (116082)

33 29 or 30 or 31 or 32 (346812)

34 26 and 33 (160)

35 27 or 28 or 34 (371)

36 exp animal/ not human.sh. (4686333)

37 27 or 28 or 34 (371)

38 exp animal/ not human.sh. (4686333)

39 37 not 38 (368)

40 39 not (letter or editorial).pt. (362)

41 remove duplicates from 40 (351)

********************

Database: Ovid MEDLINE(R) Epub Ahead of Print and In-Process & Other Non-Indexed Citations <November 22, 2019>

Search Strategy:

--------------------------------------------------------------------------------

1 (corticosteroid* or betamethasone or celestone or dexamethasone).ti,ab. (14359)

2 (adrenal cortex adj hormone*).ti,ab. (5)

3 1 or 2 (14364)

4 (prematur$ adj3 (birth$ or born or deliver$)).ti,ab. (1355)

5 (preterm or preterms or pre term or pre terms).ti,ab. (8818)

6 (preemie* or premie or premies).ti,ab. (12)

7 (late preterm or near term or almost term or before term or early term).ti,ab. (922)

8 5 or 6 or 7 (9364)

9 (newborn* or new born* or newly born* or baby or babies or infan*).ti,ab. (51878)

10 (neonat$ or neo nat$).ti,ab. (23379)

11 (antenatal or ante natal).ti,ab. (4611)

12 9 or 10 or 11 (67572)

13 3 and 8 and 12 (213)

14 "randomi?ed controlled trial".ti,ab,kw. (17005)

15 (Phase 3 or phase3 or phase iii or P3 or Piii).ti,ab,kw. (8844)

16 "randomi?ed controlled trial".ti,ab,kw. (17005)

17 RCT.ti,ab. (4115)

18 (random$ adj control$).ti,ab. (36816)

19 14 or 15 or 16 or 17 or 18 (46688)

20 13 and 19 (15)

********************

Web of Science Core Collection

| # 6 | [**24**](https://apps-webofknowledge-com.smhslibresources.health.wa.gov.au/summary.do?product=WOS&doc=1&qid=39&SID=E4rgt8YB9HW3fCmLwVU&search_mode=CombineSearches&update_back2search_link_param=yes) | #5 AND #4 AND #1  *Indexes=SCI-EXPANDED, SSCI, A&HCI, ESCI Timespan=All years* | [Edit](https://apps-webofknowledge-com.smhslibresources.health.wa.gov.au/WOS_AdvancedSearch_input.do?product=WOS&SID=E4rgt8YB9HW3fCmLwVU&search_mode=AdvancedSearch&replaceSetId=6&editState=init) |  |  |
| --- | --- | --- | --- | --- | --- |
|  | | | | | |
| # 5 | [**407,822**](https://apps-webofknowledge-com.smhslibresources.health.wa.gov.au/summary.do?product=WOS&doc=1&qid=31&SID=E4rgt8YB9HW3fCmLwVU&search_mode=AdvancedSearch&update_back2search_link_param=yes) | TS=((random* control* trial) OR RCT)  *Indexes=SCI-EXPANDED, SSCI, A&HCI, ESCI Timespan=All years* | [Edit](https://apps-webofknowledge-com.smhslibresources.health.wa.gov.au/WOS_AdvancedSearch_input.do?product=WOS&SID=E4rgt8YB9HW3fCmLwVU&search_mode=AdvancedSearch&replaceSetId=5&editState=init) |  |  |
|  | | | | | |
| # 4 | [**4,535**](https://apps-webofknowledge-com.smhslibresources.health.wa.gov.au/summary.do?product=WOS&doc=1&qid=18&SID=E4rgt8YB9HW3fCmLwVU&search_mode=CombineSearches&update_back2search_link_param=yes) | #3 AND #2  *Indexes=SCI-EXPANDED, SSCI, A&HCI, ESCI Timespan=All years* | [Edit](https://apps-webofknowledge-com.smhslibresources.health.wa.gov.au/WOS_AdvancedSearch_input.do?product=WOS&SID=E4rgt8YB9HW3fCmLwVU&search_mode=AdvancedSearch&replaceSetId=4&editState=init) |  |  |
|  | | | | | |
| # 3 | [**510,550**](https://apps-webofknowledge-com.smhslibresources.health.wa.gov.au/summary.do?product=WOS&doc=1&qid=17&SID=E4rgt8YB9HW3fCmLwVU&search_mode=AdvancedSearch&update_back2search_link_param=yes) | TS=(antenatal OR neonat* OR infant* OR newborn)  *Indexes=SCI-EXPANDED, SSCI, A&HCI, ESCI Timespan=All years* | [Edit](https://apps-webofknowledge-com.smhslibresources.health.wa.gov.au/WOS_AdvancedSearch_input.do?product=WOS&SID=E4rgt8YB9HW3fCmLwVU&search_mode=AdvancedSearch&replaceSetId=3&editState=init) |  |  |
|  | | | | | |
| # 2 | [**19,175**](https://apps-webofknowledge-com.smhslibresources.health.wa.gov.au/summary.do?product=WOS&doc=1&qid=16&SID=E4rgt8YB9HW3fCmLwVU&search_mode=AdvancedSearch&update_back2search_link_param=yes) | TS=(late NEAR/2 preterm OR "near term" OR before NEAR/2 term OR almost NEAR/2 term OR early NEAR/2 term )  *Indexes=SCI-EXPANDED, SSCI, A&HCI, ESCI Timespan=All years* | [Edit](https://apps-webofknowledge-com.smhslibresources.health.wa.gov.au/WOS_AdvancedSearch_input.do?product=WOS&SID=E4rgt8YB9HW3fCmLwVU&search_mode=AdvancedSearch&replaceSetId=2&editState=init) |  |  |
|  | | | | | |
| # 1 | [**33,949**](https://apps-webofknowledge-com.smhslibresources.health.wa.gov.au/summary.do?product=WOS&doc=1&qid=11&SID=E4rgt8YB9HW3fCmLwVU&search_mode=AdvancedSearch&update_back2search_link_param=yes) | TI=(corticosteroid* OR "adrenal cortex hormones" OR celestone OR betamethasone OR dexamethasone OR antenatal NEAR/2 steroids)  *Indexes=SCI-EXPANDED, SSCI, A&HCI, ESCI Timespan=All years* |  |  |  |

**Google Scholar**

(+corticosteroid* OR +celestone OR +betamethasone OR +dexamethasone OR +"antenatal steroids" OR +steroids)+"late preterm"

Also used keywords: premature, neonate, neonatal, fetal, foetal, infant, newborn, preterm, early term, before term

Key to symbols and search terms

* = wildcard

/ = indexed term term

exp = explodes the term entered and retrieves records that contain the term and any of its narrower, more specific terms.

mp = title, abstract, original title, name of substance word, subject heading word, keyword heading word, protocol supplementary concept word, rare disease supplementary concept word, unique identifier, synonyms]
