# Supplementary material for: Antenatal corticosteroids for impending late preterm (34-36+6 weeks) deliveries—A systematic review and meta-analysis of RCTs
Source: PLoS One. 2021 Mar 22;16(3):e0248774. doi: 10.1371/journal.pone.0248774 (PMC7984612; doi:10.1371/journal.pone.0248774)
Supplement: Attachment — Submitted filename: Reviewers response Final.docx [file pone.0248774.s013.docx]

Dear Editor

We appreciate the opportunity to revise our manuscript based on the reviewer’s comments. Please find the point-by-point response as follows. Hope to hear from you soon.

Dr Mangesh Deshmukh

Dr Sanjay Patole

**Reviewer #1:**

A) The authors responded clearly in their response to the comments on page 2 however for clarity in the 1)abstract, 2)manuscript and in the 3)tables, please add to “need for resuscitation at birth” adding “only in the delivery room immediately after birth (Not in NICU).

Response: Done;

**Abstract: Page 2, Line 35,36;**

Manuscript: **Material and methods: Page 6, line 115-16, Results: Page 12, lines 226-27,**

**Table 2**

B) Please also put back(I know it had been removed witt TC, but I did not request this removal and it is important) in your conclusion of the abstract that large definitive trials are required to assess benefits and risks of ANC in this population

Response: Thanks for the comment. The above sentence is already there in the **abstract (Page 2,3, Line 47-49)**

*Large definitive trials with adequate follow up for neurodevelopmental outcomes are required to assess benefits and risks of ANC in this population*.

C) **Please note that in track changes line 384 states “the concerns about using Apgar scores as a primary outcome need to be discussed“ please remove the sentence as this was my direction to you**

Response: We used the sentence as a linking sentence to introduce discussion on APGAR score.

We have modified the sentence as follows, in **Discussion section Page 19, line 364.**

*The limitations of using Apgar scores as a primary outcome need to be discussed.*
